# Recurring Trans-Atlantic Incursion of Clade 2.3.4.4b H5N1 Viruses by Long Distance Migratory Birds from Northern Europe to Canada in 2022/2023

**DOI:** 10.3390/v15091836

**Published:** 2023-08-30

**Authors:** Tamiru N. Alkie, Alexander M. P. Byrne, Megan E. B. Jones, Benjamin C. Mollett, Laura Bourque, Oliver Lung, Joe James, Carmencita Yason, Ashley C. Banyard, Daniel Sullivan, Anthony V. Signore, Andrew S. Lang, Meghan Baker, Beverly Dawe, Ian H. Brown, Yohannes Berhane

**Affiliations:** 1 National Centre for Foreign Animal Disease, Canadian Food Inspection Agency, Winnipeg, MB R3E 3R2, Canada; tamiru.alkie@inspection.gc.ca (T.N.A.); oliver.lung@inspection.gc.ca (O.L.); daniel.sullivan@inspection.gc.ca (D.S.); anthony.signore@inspection.gc.ca (A.V.S.); 2 Department of Virology, Animal and Plant Health Agency (APHA-Weybridge), Woodham Lane, 10 Addlestone, Surrey KT15 3NB, UK; alexander.byrne@apha.gov.uk (A.M.P.B.); benjamin.mollett@apha.gov.uk (B.C.M.); joe.james@apha.gov.uk (J.J.); ashley.banyard@apha.gov.uk (A.C.B.); 3 Canadian Wildlife Health Cooperative, Atlantic Region, Charlottetown, PE C1A 4P3, Canada; mjones@cwhc-rcsf.ca (M.E.B.J.); lbourque@cwhc-rcsf.ca (L.B.); 4 Atlantic Veterinary College, University of Prince Edward Island, Charlottetown, PE C1A 4P3, Canada; yason@upei.ca; 5 WOAH/FAO International Reference Laboratory for Avian Influenza, Animal and Plant Health 12 Agency (APHA-Weybridge), Woodham Lane, Addlestone, Surrey KT15 3NB, UK; 6 Department of Biology, Memorial University of Newfoundland, St. John’s, NL A1C 5S7, Canada; aslang@mun.ca; 7 Animal Health Division, Department of Fisheries, Forestry and Agriculture, Government of Newfoundland and Labrador, Provincial Agriculture Building, 204 Brookfield Road, St. John’s, NL A1E 0B2, Canada; meghanbaker@gov.nl (M.B.); beverlydawe@gov.nl.ca (B.D.); 8 Department of Veterinary Pathology, Western College of Veterinary Medicine, University of Saskatchewan, Saskatoon, SK S7N 5B4, Canada; 9 Department of Animal Science, University of Manitoba, Winnipeg, MB R3T 2N2, Canada

**Keywords:** H5N1, clade 2.3.4.4b, American crow, red fox, Canada, re-introduction

## Abstract

In December 2022 and January 2023, we isolated clade 2.3.4.4b H5N1 high-pathogenicity avian influenza (HPAI) viruses from six American crows (*Corvus brachyrhynchos*) from Prince Edward Island and a red fox (*Vulpes vulpes*) from Newfoundland, Canada. Using full-genome sequencing and phylogenetic analysis, these viruses were found to fall into two distinct phylogenetic clusters: one group containing H5N1 viruses that had been circulating in North and South America since late 2021, and the other one containing European H5N1 viruses reported in late 2022. The transatlantic re-introduction for the second time by pelagic/Icelandic bird migration via the same route used during the 2021 incursion of Eurasian origin H5N1 viruses into North America demonstrates that migratory birds continue to be the driving force for transcontinental dissemination of the virus. This new detection further demonstrates the continual long-term threat of H5N1 viruses for poultry and mammals and the subsequent impact on various wild bird populations wherever these viruses emerge. The continual emergence of clade 2.3.4.4b H5Nx viruses requires vigilant surveillance in wild birds, particularly in areas of the Americas, which lie within the migratory corridors for long-distance migratory birds originating from Europe and Asia. Although H5Nx viruses have been detected at higher rates in North America since 2021, a bidirectional flow of H5Nx genes of American origin viruses to Europe has never been reported. In the future, coordinated and systematic surveillance programs for HPAI viruses need to be launched between European and North American agencies.

## 1. Introduction

Waterfowls (*Anseriformes*) and shorebirds (*Charadriiformes*) are regarded as the major vectors for the propagation of both low-pathogenicity (LP) and high-pathogenicity (HP) avian influenza (AI) viruses as well as in the dissemination of these viruses to a wide range of avian species en route to breeding and wintering sites [1]. The GsGd (Goose/Guangdong) lineage of H5 HPAI viruses has steadily evolved into genetically and antigenically distinct clades with varying degrees of pathogenicity for avian hosts in the past 25 years [2,3]. An elevated frequency of H5 HPAI outbreaks in wild birds and gallinaceous poultry have caused mass mortality events and the culling of millions of poultry globally, resulting in significant economic losses. In Europe, clade 2.3.4.4a H5N8 HPAI viruses of Eurasian origin that were introduced by migratory birds in autumn 2014 had caused limited epizootics. However, the introduction of clade 2.3.4.4b H5N8 HPAI viruses by wild birds in 2016 inflicted major widespread outbreaks in poultry and wild birds during winter 2016/2017 in Europe. Subsequently, in autumn 2020, clade 2.3.4.4b H5N8 HPAI viruses re-emerged in Europe and spread across the northern hemisphere, causing severe outbreaks in the autumn and winter months of 2020/2021 [4,5]. Interspersed between the two major outbreaks that occurred in 2016/2017 and 2020/2021 was the detection of multisubtype H5Nx viruses causing local and sporadic epizootics at a smaller scale [5,6,7].

In 2021/2022 clade 2.3.4.4b H5N1 HPAI virus, a reassortant virus derived from H5N8 virus during the 2020/2021 epizootic, appeared in Europe and has dominated over all other subtypes, causing widespread epizootic diseases that has caused mass mortality events across several wild bird species with frequent spillover infections into mammals [8,9,10,11]. The significantly higher HPAI infection pressure in wild birds inhabiting all major regional and intercontinental wild bird migratory pathways has then resulted in further spread and generation of reassortant viruses [11,12,13]. The 2021 incursion of clade 2.3.4.4b H5N1 virus into Canada for the first time in almost a decade after detection of clade 2.3.4.4c H5N8 and its reassortant H5N2 and H5N1 subtypes in 2014, is an impeccable example of how infection pressure in Eurasia can contribute to the global dissemination of HPAI viruses [9]. The incursion into Canada was first confirmed in December 2021 from avian samples collected in St. John’s, Newfoundland and Labrador. Initially, species affected included silky chickens, guinea fowl, peafowl, emus, domestic ducks, geese and turkeys on an exhibition farm, and free-living great black-backed gulls (*Larus marinus*) but dissemination beyond the initial outbreaks occurred within a few months [9]. Incursions were also reported around the same time in the USA, whereby the virus was confirmed in hunter-collected American wigeons (*Mareca americana*) and with subsequent outbreaks being detected in domestic poultry along the east coast of the country [8]. The pre-emptive responses of restricting domestic poultry movements in and out of infected zones and poultry flock depopulation in infected and contact premises have cost the poultry industry billions of US dollars. Currently, the viruses are quickly spreading and have been detected in many wild waterfowl and shorebird species in the Americas. In addition, H5N1 HPAI viruses have inflicted mass die-offs in raptors, scavenger birds and several terrestrial and aquatic mammalian species. In one of the ecologically vital species in North America alone, the bald eagle (*Haliaeetus leucocephalus*), nest failure attributed to the death of breeding adults and nestlings due to H5N1 HPAI was significant [14]. In winter 2022, new threats and second primary incursions of H5N1 HPAI viruses were reported on the west coast of Canada in British Columbia. Phylogenetically, these viruses were found to be highly related to two viruses isolated in wild birds and domestic chickens from Japan in January 2022 [15].

Starting from the summer/autumn of 2021 and spring 2022, two lineages of clade 2.3.4.4b H5N1 viruses, designated as B1 and B2 based on the HA gene, were found to be independently carried into Iceland from Europe by migratory birds [12]. As the main route of H5Nx HPAI virus incursion, migratory birds carried the virus from Europe to Iceland/Greenland and further south migration in fall introduced the virus to both Canada and the USA [8,9]. Phylogenetic analysis supported this hypothesis as highly genetically similar viruses were found in Iceland, Europe and North America. Recent efforts to unravel the means of incursions into North America by retrospectively analyzing leg band data have shown that long-distance migratory aquatic birds from Eurasia primarily utilize the transatlantic [9] and also the Pacific routes [15] to move between ecological niches following seasonal changes [9]. Long-range pelagic movements of birds could also potentially disseminate the virus from Europe to North America. The hosts implicated as the major contributors for continuous virus evolution, generation of reassortant viruses and persistence in various ecologies, and dissemination beyond geographical borders are likely geese, dabbling ducks, seabirds and shorebirds.

The current study describes seven HPAI viruses belonging to clade 2.3.4.4b H5N1 virus isolated from dead American crows (*Corvus brachyrhynchos*) and a red fox (*Vulpes vulpes*) from Eastern Canada. Five of the viruses were reassortant viruses related to those already circulating in North America since 2021, but the other two isolates were wholly Eurasian H5N1 viruses associated with new incursions into Canada from Northern Europe, mediated by bird migration. These findings demonstrate that early surveillance of the virus in migratory birds along the Pacific and Atlantic flyways before active migration seasons begin can help define the risks these viruses pose to poultry and wildlife.

## 2. Materials and Methods

### 2.1. Clinical Samples

Brian tissues were collected from six dead American crows (*Corvus brachyrhynchos*) between 26 and 28 December 2022 in Prince Edward Island (PEI) and a red fox (*Vulpes vulpes*) in January 2023 from Newfoundland. The samples were tested in a provincial lab for influenza A viruses and submitted to the National Centre for Foreign Animal Disease (NCFAD), Winnipeg, Manitoba, for confirmatory testing. Total RNA was extracted from all brain tissue samples using the MagMax 1836 Nucleic Acid Isolation Kit using the KingFisher Duo prime platform (ThermoFisher Scientific, Waltham, MA, USA). As in previous studies, influenza A virus nucleic acid was detected initially using the matrix (M) gene and followed by H5 and H7 gene-specific real-time RT-PCR testing [16,17]. Moreover, viable viruses were isolated in specific pathogen-free embryonated chicken eggs as previously described [18].

### 2.2. Nanopore Sequencing and Genome Assembly

From clinical specimens and isolated viruses, the whole genome segments of IAVs were amplified by one step RT-PCR as described previously [19]. The Nanopore sequencing method was performed on an Oxford Nanopore GridION sequencer with an R9.4.1 flow cell after library construction using a rapid barcoding kit (SQK-RBK004 or SQK-RBK110.96). The sequence data obtained were treated as described previously [15].

### 2.3. Phylogenetic Analysis

H5N1 whole-genome sequences originating from Europe, North America and South America between 1 October 2021, through to 24 February 2023, and deposited in the Global Initiative on Sharing All Influenza Data (GISAID) were used in our analysis. To these sequences, we added the seven sequences generated from samples collected from Prince Edward Island (PEI) and Newfoundland, Canada (Table 1), as well as four unpublished avian H5N1 sequences from the United Kingdom, generated by the Animal and Plant Health Agency, UK (Table S1). Sequences were aligned using MAFFT v7.487 [20] and manually trimmed to the open reading frame using AliView. Phylogenetic trees were then inferred using the maximum-likelihood approach in IQ-Tree v2.1.4 [21] with ModelFinder [22] to infer the appropriate phylogenetic model and using 1000 ultrafast bootstraps [23]. Ancestral sequence reconstruction and inference of molecular-clock phylogenies were performed using TreeTime [24]. Phylogenetic trees were visualized using R version 4.1.1, with libraries ggplot2, ggtree [25] and treeio [26]. Times for the most recent common ancestor (TMRCA) were calculated in TreeTime.

## 3. Results and Discussion

Since 2020, Clade 2.3.4.4b H5N1 HPAI outbreaks have affected a wider range of avian species, including birds of prey and scavenger species, than previous epizootics. The 2014/2015 HPAI outbreaks in Canada were caused by a reassortant clade 2.3.4.4c H5N2 HPAI virus [27] that arose from an East Asian H5N8 HPAI virus introduced via the Beringian Crucible before spilling into wild birds of the Pacific and central flyways [28]. These viruses were detected in a small range of wild birds, rarely in birds of prey or scavenger birds, and none were detected in mammals [29]. The typical characteristics of clade 2.3.4.4b H5N1 infection in scavenger birds including American crows and in mammals are manifested in the form of neurological disease and hyper-acute mortality [30,31,32].

**Figure 1:**
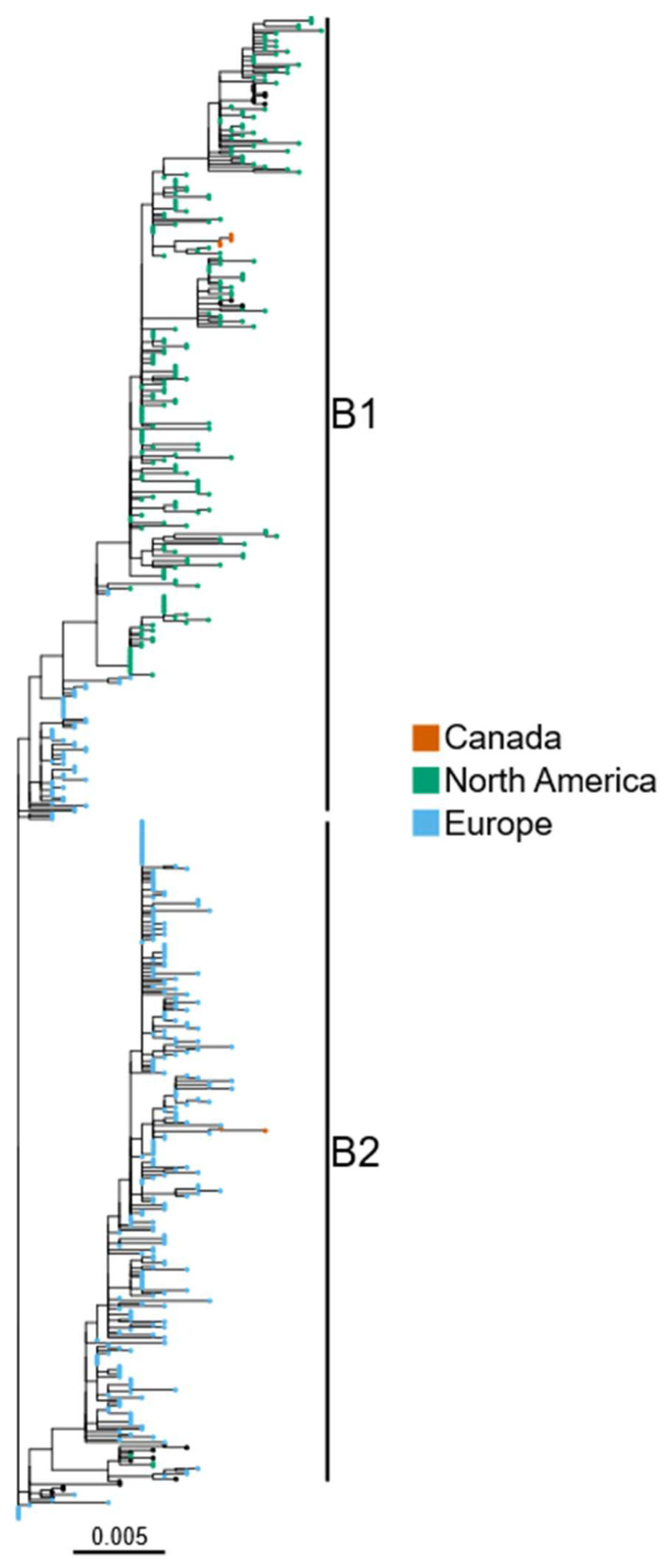
H5N1 HPAI viruses from Canada belong to two distinct phylogenetic groups. H5N1 HA sequences from North and South America, Europe, Asia and Africa collected since October 2021 were used to generate a maximum-likelihood phylogenetic tree. Sequences from North America and Europe generated in this study are highlighted by brown colors, and the B1 and B2 HA sub-lineages [5] are also labelled. Sequences from other geographical locations have black node tips.

In December 2022, six American crows were found dead on Prince Edward Island, Canada. In January 2023, a red fox was also found dead in Newfoundland, Canada. Brain tissue samples were collected and tested for the presence of HPAI viruses. All samples were found to be positive for the presence of H5N1 HPAI virus, and whole-genome sequencing (WGS) was performed. To confirm the origin and the time of introduction of these viruses, phylogenetic analyses were performed using contemporary H5N1 sequences from North and South America, Europe, Asia and Africa. All seven Canadian H5N1 HA sequences were found to belong to clade 2.3.4.4b viruses. One of the sequences obtained from an American crow (A/American crow/PEI/FAV-0019-01/2022) and the sequence obtained from a red fox (A/Red fox/NL/FAV-0075/2023) were clustered with the European H5N1 viruses belonging to the B2 HA sub-lineage [5] (Figure 1) and are hereafter referred to as Group 1. Across the other gene segments (PB2, PB1, PA, NP, NA, M and NS), the sequences of these two viruses were also found to cluster with the European sequences (Figure S1), with the closest relative (>97% nucleotide identity compared to Group 1 sequences across all gene segments) being a sequence obtained from a poultry outbreak on the Orkney Islands, Great Britain, from October 2022. The dates for the most recent common ancestor of the Group 1 sequences to which the two recent H5N1 viruses belong range from October 2022 to December 2022 (lower bound: May 2022, higher bound: December 2022) (Table 2). The avian species that most likely play a role in the transfer of the virus from Europe to Eastern Canada, potentially via Icelandic/pelagic routes, were discussed in our previous study [9]. However, a number of intercontinental migratory birds could contribute to such long-distance dissemination. Several wild bird species including gulls are important in spreading viruses to ducks, geese and scavenger birds [9,15].

The remaining five H5N1 sequences generated in the current study from American crows (referred to as Group 2 hereafter) were clustered with North and South American H5N1 sequences within the B1 lineage for the HA gene (Figure 1). The same clustering pattern was observed for the other gene segments (Figure S1).

This study represents the first report on the presence of H5N1 HPAI viruses with different genome constellations/reassortment patterns in Prince Edward Island of Canada since the initial introduction of the H5N1 virus in late 2021. During the 2021/2022 HPAI outbreaks in Canada, H5N1 reassortant viruses were first reported in central and Mississippi flyways in Canada in the spring of 2022 which then disseminated widely. The reassortant viruses identified in the American crows in Prince Edward Island in December 2022 contain PB2, PB1, NP and NS segments from unidentified North American lineage avian influenza viruses that have been circulating in North American wild birds and the other four segments from Eurasian viruses described in our previous study [30]. To date, we were able to identify clade 2.3.4.4b H5N1 viruses with different patterns of genome constellations involving Eurasian and North American lineage viruses in all regions and territories of Canada, with the exception of Newfoundland and Labrador where all H5N1 viruses characterized so far are entirely of Eurasian origin [30]. Reassortant H5N1 viruses with PB2, PB1, NP and NS segments from North American lineage H5N1 viruses were initially predominantly detected in central and, to a lesser extent, Pacific Mississippi wild bird flyways in spring/summer of 2022. However, starting from fall of 2022, we started to observe a continued geographical expansion of viruses with the same genome constellation pattern in the Atlantic and Mississippi flyways.

The patterns of relationships of gene segments within European viruses forced us to explore the connection between migratory wild bird flyways and virus maintenance. The higher infection pressure and detection rate of H5N1 HPAI viruses in wild birds in Western Europe in all seasons from 2021 to 2022 possibly enhanced the dissemination of the virus to birds migrating mainly to Iceland. The Atlantic flyway, which is the prominent flyway connecting Europe and Canada via Iceland or Greenland, was regarded as the means of introduction of emerging H5N1 viruses to the most eastern parts of Canada and the USA before dissemination to other provinces or states [9]. Starting from the summer/autumn of 2021 and spring 2022, two sub-lineages of clade 2.3.4.4b H5N1 viruses, designated as B1 and B2 based on HA gene clustering, were independently introduced into Iceland from Europe by migratory birds [12]. Previous works that analyzed banding records collected from migratory birds showed that a vast majority of migratory waterfowls can migrate as far as to Iceland and Greenland due to seasonal changes and hence can translocate viruses from congregation sites and likely contribute to the intercontinental spread of H5N1 HPAI viruses. The re-introduction of new viruses and recurring local expansions of divergent or reassortant HPAI viruses within the East Coast of Canada and the USA could impact the genetic landscape within the North American flyways. Moreover, the continued movements of birds through the North American flyways that consist of three hotspots for avian influenza virus, the Delaware Bay for shorebirds and gulls, Alberta for ducks, and Chesapeake Bay, wherein millions of birds congregate, may have long-term consequences for virus diversity and new variant generation [33,34].

Here, we demonstrated a new incursion of a genetically distinct H5N1 HPAI virus into Eastern Canada during late 2022, representing a second incursion into this region after clade 2.3.4.4b HPAIV first arrived in North America in late 2021. On both occasions, the most parsimonious explanation is that the virus had been translocated by migratory birds from Northern Europe via the transatlantic route. The continual emergence of clade 2.3.4.4b viruses in avian hosts [35] requires vigilant surveillance for avian influenza in wild birds, particularly in areas of the Americas that are entry points for long-distance migratory birds from Europe and Asia. This complex and intertwining inter-continental seasonal connectivity of wild birds has led to the introduction of H5Nx HPAI viruses to Canada four times so far during 2014/2015, late 2021, winter/spring 2022 and winter 2022/2023, and raises the possibility that this could become a regular occurrence if these viruses remain circulating in wilds birds. To date, this flow of viruses has only been detected from Europe to North America, but the continued circulation of these viruses in wild birds and their increasing expansion in host ranges (including new bird taxa) creates increased opportunity for the spread of the virus through multiple wild bird migratory pathways, both short and long. The possibility of bi-directional translocation of viruses is highly plausible given the known wild bird movement pathways (albeit smaller in scale than north to south routes) and considering the genetic diversity, increasing the likelihood that viruses which have evolved independently in the Americas could be detected on the eastern Atlantic seaboard. This level of risk reinforces the need for enhanced genetic surveillance, which is crucial to identifying such occurrences.

## Figures and Tables

**Table 1:** H5N1 HPAI virus sequences from Canada generated in this study.

Sequences	Collection Date	Species	Location	Phylogenetic Group	GISAID EpiFlu Accession Number
A/Red fox/NL/FAV-0075/2023	31 January 2023	Red fox	Newfoundland	1	EPI_ISL_17494318
A/American crow/PEI/FAV-0019-01/2022	26 December 2022	American crow	Prince Edward Island	1	EPI_ISL_17494319
A/American crow/PEI/FAV-0019-02/2022	28 December 2022	American crow	Prince Edward Island	2	EPI_ISL_17478907
A/American crow/PEI/FAV-0019-03/2022	28 December 2022	American crow	Prince Edward Island	2	EPI_ISL_17479113
A/American crow/PEI/FAV-0019-04/2022	28 December 2022	American crow	Prince Edward Island	2	EPI_ISL_17479167
A/American crow/PEI/FAV-0019-05/2022	28 December 2022	American crow	Prince Edward Island	2	EPI_ISL_17479251
A/American crow/PEI/FAV-0019-06/2022	28 December 2022	American crow	Prince Edward Island	2	EPI_ISL_17479396

**Table 2:** Dates for the most recent common ancestor (MRCA) of all gene segments for the recent Canadian Group 1 and 2 H5N1 virus sequences.

Segment	Group 1			Group 2		
	Node Date	Lower Bound	Higher Bound	Node Date	Lower Bound	Higher Bound
PB2	22 December 2022	12 October 2022	26 December 2022	24 August 2022	2 June 2022	31 October 2022
PB1	12 October 2022	6 August 2022	12 October 2022	28 December 2022	18 October 2022	28 December 2022
PA	9 November 2022	22 September 2022	20 December 2022	29 October 2022	10 September 2022	2 December 2022
HA	28 October 2022	28 August 2022	22 December 2022	28 December 2022	2 November 2022	28 December 2022
NP	12 October 2022	12 July 2022	12 October 2022	31 August 2022	11 June 2022	11 November 2022
NA	24 November 2022	27 August 2022	21 December 2022	22 June 2022	15 March 2022	6 October 2022
MP	26 December 2022	23 July 2022	26 December 2022	2 February 2022	11 September 2021	12 August 2022
NS	10 October 2022	4 May 2022	12 October 2022	27 July 2022	26 March 2022	27 November 2022

## Data Availability

The sequences generated from the current study were deposited in the Global Initiative on Sharing All Influenza Data (GISAID) EpiFlu™ database.

## Supplementary Materials

The following supporting information can be downloaded at: https://www.mdpi.com/article/10.3390/v15091836/s1, Table S1. Additional H5N1 HPAI virus sequences from the United Kingdom used in this study. Figure S1. H5N1 sequences from North and South America, Europe, Asia and Africa collected since October 2021 were used to generate maximum-likelihood phylogenetic trees for the HA, NA, PB2, PB1, PA, NP, MP and NS gene segments. Sequences from North America, Europe and the sequences generated in this study are highlighted, and the B1 and B2 HA sub-lineages [5] are also shown in the HA tree.

## References

[1] Hill N.J., Bishop M.A., Trovão N.S., Ineson K.M., Schaefer A.L., Puryear W.B., Zhou K., Foss A.D., Clark D.E., MacKenzie K.G. (2022). Ecological Divergence of Wild Birds Drives Avian Influenza Spillover and Global Spread. PLoS Pathog..

[2] Xu X., Subbarao K., Cox N.J., Guo Y. (1999). Genetic Characterization of the Pathogenic Influenza A/Goose/Guangdong/1/96 (H5N1) Virus: Similarity of its Hemagglutinin Gene to those of H5N1 Viruses from the 1997 Outbreaks in Hong Kong. Virology.

[3] Chen H., Smith G.J., Li K.S., Wang J., Fan X.H., Rayner J.M., Vijaykrishna D., Zhang J.X., Zhang L.J., Guo C.T. (2006). Establishment of Multiple Sublineages of H5N1 Influenza Virus in Asia: Implications for Pandemic Control. Proc. Natl. Acad. Sci. USA.

[4] Lin R., Lu L., Lycett S., Liu W., Li J. (2021). Dealing with Highly Pathogenic Avian Influenza: An Impending Crisis. Innovation.

[5] Pohlmann A., King J., Fusaro A., Zecchin B., Banyard A.C., Brown I.H., Byrne A.M.P., Beerens N., Liang Y., Heutink R. (2022). Has Epizootic Become Enzootic? Evidence for a Fundamental Change in the Infection Dynamics of Highly Pathogenic Avian Influenza in Europe, 2021. mBio.

[6] Beerens N., Koch G., Heutink R., Harders F., Vries D.P.E., Ho C., Bossers A., Elbers A. (2018). Novel Highly Pathogenic Avian Influenza A(H5N6) Virus in the Netherlands, December 2017. Emerg. Infect. Dis..

[7] Śmietanka K., Świętoń E., Kozak E., Wyrostek K., Tarasiuk K., Tomczyk G., Konopka B., Welz M., Domańska-Blicharz K., Niemczuk K. (2020). Highly Pathogenic Avian Influenza H5N8 in Poland in 2019-2020. J. Vet. Res..

[8] Bevins S.N., Shriner S.A., Cumbee J.C., Dilione K.E., Douglass K.E., Ellis J.W., Killian M.L., Torchetti M.K., Lenoch J.B. (2022). Intercontinental Movement of Highly Pathogenic Avian Influenza A(H5N1) Clade 2.3.4.4 Virus to the United States, 2021. Emerg. Infect. Dis..

[9] Caliendo V., Lewis N.S., Pohlmann A., Baillie S.R., Banyard A.C., Beer M., Brown I.H., Fouchier R.A.M., Hansen R.D.E., Lameris T.K. (2022). Transatlantic Spread of Highly Pathogenic Avian Influenza H5N1 by Wild Birds from Europe to North America in 2021. Sci. Rep..

[10] Rijks J.M., Leopold M.F., Kühn S., In‘t Veld R., Schenk F., Brenninkmeijer A., Lilipaly S.J., Ballmann M.Z., Kelder L., de Jong J.W. (2022). Mass Mortality Caused by Highly Pathogenic Influenza A(H5N1) Virus in Sandwich Terns, the Netherlands, 2022. Emerg. Infect. Dis..

[11] Lewis N.S., Banyard A.C., Whittard E., Karibayev T., Al Kafagi T., Chvala I., Byrne A., Meruyert Akberovna S., King J., Harder T. (2021). Emergence and Spread of Novel H5N8, H5N5 and H5N1 Clade 2.3.4.4 Highly Pathogenic Avian Influenza in 2020. Emerg. Microbes Infect..

[12] Günther A., Krone O., Svansson V., Pohlmann A., King J., Hallgrimsson G.T., Skarphéðinsson K.H., Sigurðardóttir H., Jónsson S.R., Beer M. (2022). Iceland as Stepping Stone for Spread of Highly Pathogenic Avian Influenza Virus between Europe and North America. Emerg. Infect. Dis..

[13] Verhagen J.H., Fouchier R.A.M., Lewis N. (2021). Highly Pathogenic Avian Influenza Viruses at the Wild-Domestic Bird Interface in Europe: Future Directions for Research and Surveillance. Viruses.

[14] Nemeth N.M., Ruder M.G., Poulson R.L., Sargent R., Breeding S., Evans M.N., Zimmerman J., Hardman R., Cunningham M., Gibbs S. (2023). Bald Eagle Mortality and Nest Failure Due to Clade 2.3.4.4 Highly Pathogenic H5N1 Influenza A Virus. Sci. Rep..

[15] Alkie T.N., Lopes S., Hisanaga T., Xu W., Suderman M., Koziuk J., Fisher M., Redford T., Lung O., Joseph T. (2022). A Threat from both Sides: Multiple Introductions of Genetically Distinct H5 HPAI Viruses into Canada via both East Asia-Australasia/Pacific and Atlantic Flyways. Virus Evol..

[16] Weingartl H.M., Berhane Y., Hisanaga T., Neufeld J., Kehler H., Emburry-Hyatt C., Hooper-McGreevy K., Kasloff S., Dalman B., Bystrom J. (2010). Genetic and Pathobiologic Characterization of Pandemic H1N1 2009 Influenza Viruses from a Naturally Infected Swine herd. J. Virol..

[17] Spackman E., Senne D.A., Myers T.J., Bulaga L.L., Garber L.P., Perdue M.L., Lohman K., Daum L.T., Suarez D.L. (2002). Development of a Real-Time Reverse Transcriptase PCR Assay for Type A Influenza Virus and the Avian H5 and H7 Hemagglutinin Subtypes. J. Clin. Microbiol..

[18] OIE (2021). Avian Influenza. Manual of Diagnostic Tests and Vaccines for Terrestrial Animals.

[19] Zhou B., Donnelly M.E., Scholes D.T., St George K., Hatta M., Kawaoka Y., Wentworth D.E. (2009). Single-reaction Genomic Amplification Accelerates Sequencing and Vaccine Production for Classical and Swine Origin Human Influenza A Viruses. J. Virol..

[20] Katoh K., Standley D.M. (2013). MAFFT Multiple Sequence Alignment Software Version 7: Improvements in Performance and Usability. Mol. Biol. Evol..

[21] Minh B.Q., Schmidt H.A., Chernomor O., Schrempf D., Woodhams M.D., von Haeseler A., Lanfear R. (2020). IQ-TREE 2: New Models and Efficient Methods for Phylogenetic Inference in the Genomic Era. Mol. Biol. Evol..

[22] Kalyaanamoorthy S., Minh B.Q., Wong T.K.F., von Haeseler A., Jermiin L.S. (2017). ModelFinder: Fast Model Selection for Accurate Phylogenetic Estimates. Nat. Methods.

[23] Hoang D.T., Chernomor O., von Haeseler A., Minh B.Q., Vinh L.S. (2018). UFBoot2: Improving the Ultrafast Bootstrap Approximation. Mol. Biol. Evol..

[24] Sagulenko P., Puller V., Neher R.A. (2018). TreeTime: Maximum-likelihood Phylodynamic Analysis. Virus Evol..

[25] Guangchuang Y., David K.S., Huachen Z., Yi G., Tommy T.-Y.L. (2017). GGTREE: An R Package for Visualization and Annotation of Phylogenetic Trees with Their Covariates and other Associated Data. Methods Ecol. Evol..

[26] Wang L.G., Lam T.T., Xu S., Dai Z., Zhou L., Feng T., Guo P., Dunn C.W., Jones B.R., Bradley T. (2020). An R Package for Phylogenetic Tree Input and Output with Richly Annotated and Associated Data. Mol. Biol. Evol..

[27] Pasick J., Berhane Y., Joseph T., Bowes V., Hisanaga T., Handel K., Alexandersen S. (2015). Reassortant Highly Pathogenic Influenza A H5N2 Virus Containing Gene Segments Related to Eurasian H5N8 in British Columbia, Canada, 2014. Sci. Rep..

[28] Lee D.H., Torchetti M.K., Winker K., Ip H.S., Song C.S., Swayne D.E. (2015). Intercontinental Spread of Asian-Origin H5N8 to North America through Beringia by Migratory Birds. J. Virol..

[29] Shriner S.A., Root J.J., Lutman M.W., Kloft J.M., VanDalen K.K., Sullivan H.J., White T.S., Milleson M.P., Hairston J.L., Chandler S.C. (2016). Surveillance for Highly Pathogenic H5 Avian Influenza Virus in Synanthropic Wildlife Associated with Poultry Farms during an Acute Outbreak. Sci. Rep..

[30] Alkie T.N., Cox S., Embury-Hyatt C., Stevens B., Pople N., Pybus M.J., Xu W., Hisanaga T., Suderman M., Koziuk J. (2023). Characterization of Neurotropic HPAI H5N1 Viruses with Novel Genome Constellations and Mammalian Adaptive Mutations in Free-Living Mesocarnivores in Canada. Emerg. Microbes Infect..

[31] Bordes L., Vreman S., Heutink R., Roose M., Venema S., Pritz-Verschuren S.B.E., Rijks J.M., Gonzales J.L., Germeraad E.A., Engelsma M. (2023). Highly Pathogenic Avian Influenza H5N1 Virus Infections in Wild Red Foxes (*Vulpes vulpes*) Show Neurotropism and Adaptive Virus Mutations. Microbiol. Spectr..

[32] Caliendo V., Leijten L., van de Bildt M.W.G., Fouchier R.A.M., Rijks J.M., Kuiken T. (2022). Pathology and Virology of Natural Highly Pathogenic Avian Influenza H5N8 Infection in Wild Common Buzzards (*Buteo buteo*). Sci. Rep..

[33] Hill N.J., Ma E.J., Meixell B.W., Lindberg M.S., Boyce W.M., Runstadler J.A., Bahl J., Krauss S., Kühnert D., Fourment M. (2013). Influenza A Virus Migration and Persistence in North American Wild Birds. PLoS Pathog..

[34] Prosser D.J., Chen J., Ahlstrom C.A., Reeves A.B., Poulson R.L., Sullivan J.D., McAuley D., Callahan C.R., McGowan P.C., Bahl J. (2022). Maintenance and Dissemination of Avian-Origin Influenza A Virus within the Northern Atlantic Flyway of North America. PLoS Pathog..

[35] Huang Z.Y.X., Xu C., van Langevelde F., Ma Y., Langendoen T., Mundkur T., Si Y., Tian H., Kraus R.H.S., Gilbert M. (2019). Contrasting Effects of Host Species and Phylogenetic Diversity on the Occurrence of HPAI H5N1 in European Wild Birds. J. Anim. Ecol..

