# Supplementary material for: Recurring Trans-Atlantic Incursion of Clade 2.3.4.4b H5N1 Viruses by Long Distance Migratory Birds from Northern Europe to Canada in 2022/2023
Source: Viruses. 2023 Aug 30;15(9):1836. doi: 10.3390/v15091836 (PMC10536465; doi:10.3390/v15091836)
Supplement: Supplementary file 1 [file viruses-15-01836-s001.zip › viruses-2559797-supplementary Table S1.pdf]

Table S1. Additional H5N1 HPAI virus sequences from the United Kingdom used in this study.

Sequences	Collection Dates	GISAID EpiFlu Accession Number
A/gannet/Scotland/084624/2022	2022-06-07	EPI_ISL_13969425
A/chicken/England/115126/2022	2022-09-05	EPI_ISL_15038820
A/fulmar/Scotland/118673/2022	2022-09-12	EPI_ISL_15586131
A/chicken/Scotland/130966/2022	2022-10-12	EPI_ISL_15585888
