# Supplementary figures and images for: Recurring Trans-Atlantic Incursion of Clade 2.3.4.4b H5N1 Viruses by Long Distance Migratory Birds from Northern Europe to Canada in 2022/2023
Source: Viruses. 2023 Aug 30;15(9):1836. doi: 10.3390/v15091836 (PMC10536465; doi:10.3390/v15091836)

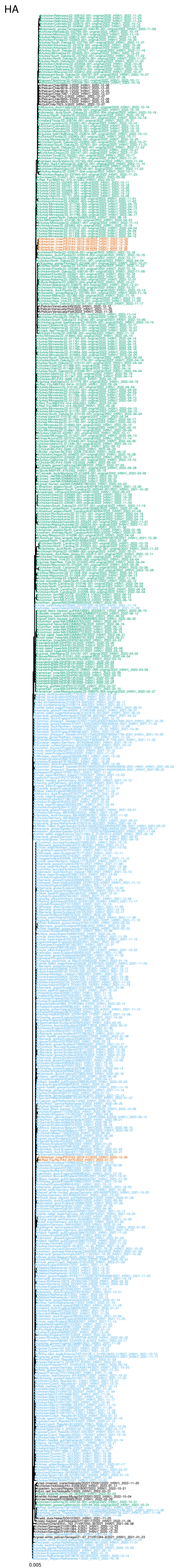

B1

B2

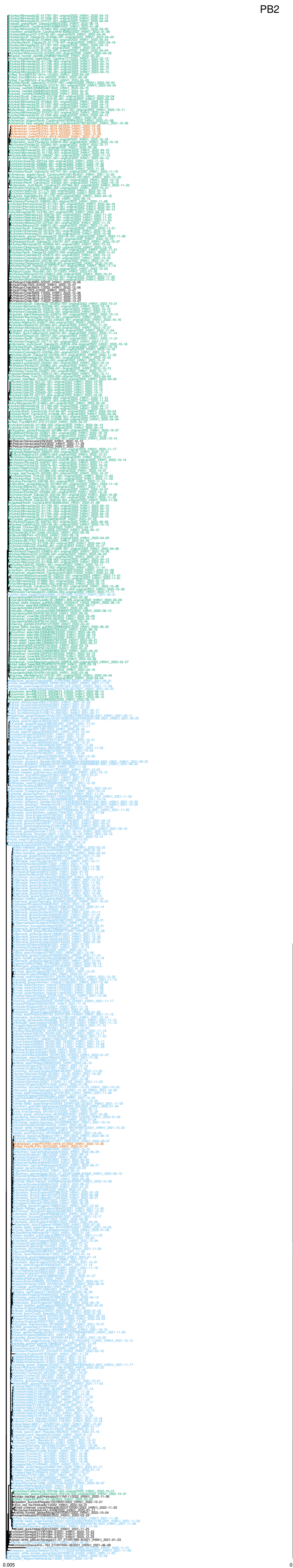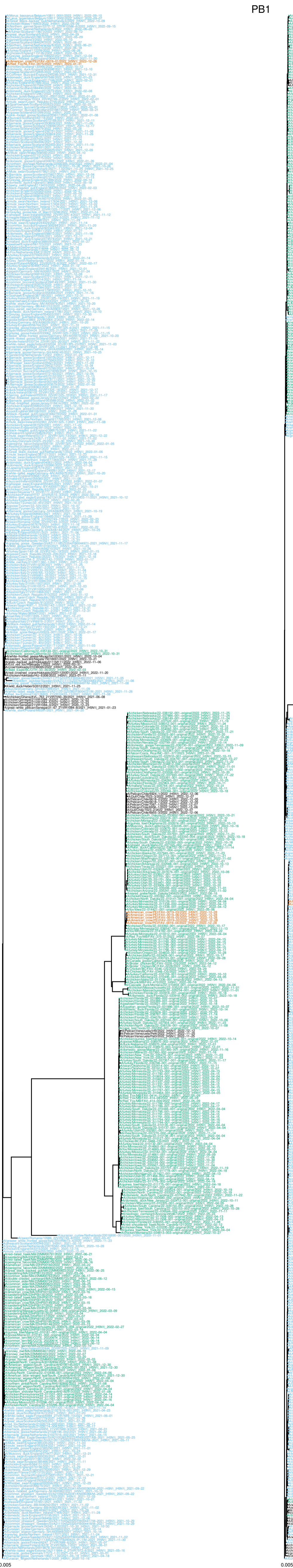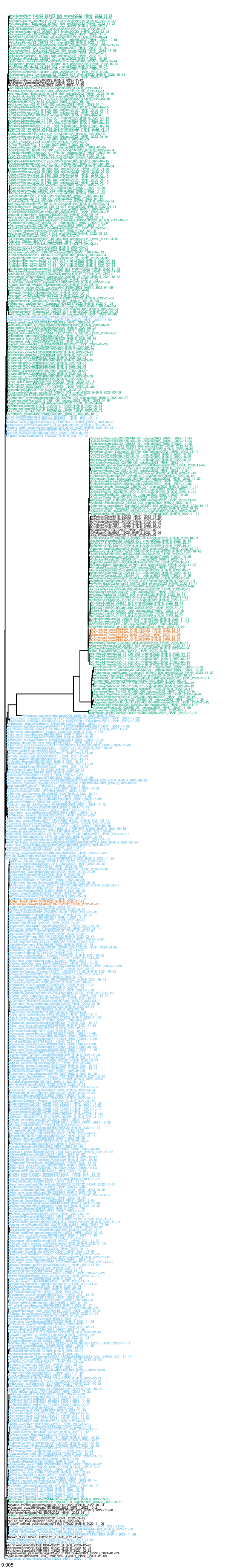

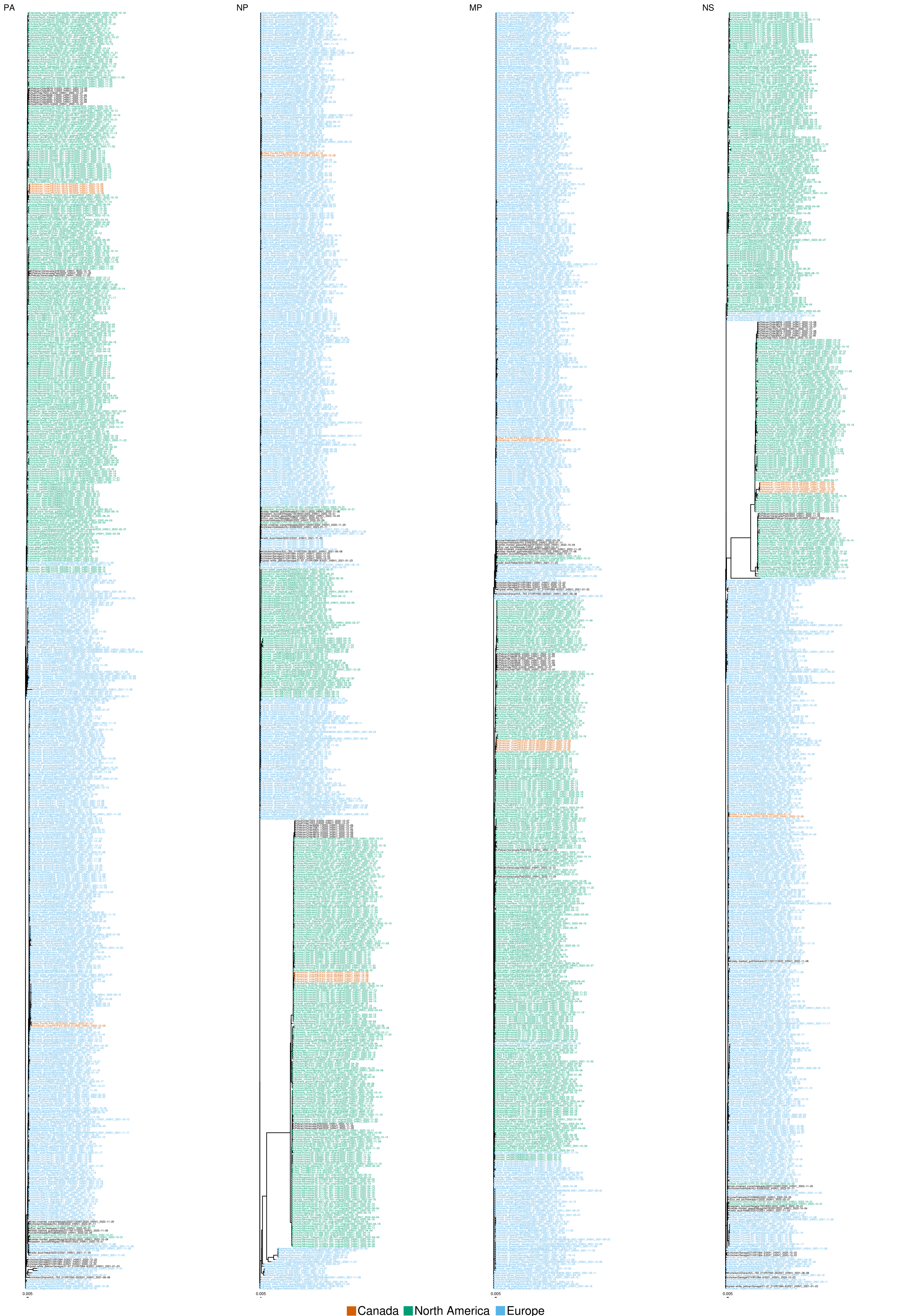

Supplement: Supplementary file 1 [file viruses-15-01836-s001.zip › viruses-2559797-supplementary Figure S1.pdf]
